# Spanish translation of the Expert Recommendations for Implementing Change (ERIC) compilation

**DOI:** 10.1186/s43058-024-00616-6

**Published:** 2024-07-17

**Authors:** Amelia E. Van Pelt, Alejandra Paniagua-Avila, Amanda Sanchez, Stephanie Sila, Elizabeth D. Lowenthal, Byron J. Powell, Rinad S. Beidas

**Affiliations:** 1 Department of Medical Social Sciences, Northwestern University Feinberg School of Medicine, 625 N Michigan Ave Suite 2100, Chicago, IL 60611 USA; 2 Center for Dissemination and Implementation Science, Northwestern University Feinberg School of Medicine, 633 N St Clair St Suite 2000, Chicago, IL 60611 USA; 3 Global Health Center, Children’s Hospital of Philadelphia, 734 Schuylkill Avenue, Philadelphia, PA 19146 USA; 4 Department of Epidemiology, Mailman School of Public Health, Columbia University, New York City, NY 10032 USA; 5 Department of Psychology, George Mason University, 4400 University Dr, Fairfax, VA 22030 USA; 6 School of Veterinary Medicine, University of Pennsylvania, 3800 Spruce Street, Philadelphia, PA 19104 USA; 7 Departments of Pediatrics and Biostatistics, Epidemiology and Informatics, University of Pennsylvania Perelman School of Medicine, 3600 Civic Center Blvd, Philadelphia, PA 19146 USA; 8 Brown School, Center for Mental Health Services Research, Washington University in St. Louis, St. Louis, MO 63130 USA; 9 Center for Dissemination and Implementation, Institute for Public Health, Washington University in St. Louis, St. Louis, MO 63110 USA; 10 Division of Infectious Diseases, John T. Milliken Department of Internal Medicine, Washington University School of Medicine in St. Louis, St. Louis, MO 63110 USA

**Keywords:** Implementation science, Implementation strategies, Translation, Global health, Spanish, ERIC compilation

## Abstract

**Background:**

Most implementation science resources (e.g., taxonomies) are published in English. Linguistic inaccessibility creates a barrier to the conduct of implementation research among non-English-speaking populations, so translation of resources is needed. Translation into Spanish can facilitate widespread reach, given the large proportion of Spanish speakers around the world. This research aimed to systematically translate the Expert Recommendations for Implementation Change (ERIC) compilation into Spanish as an exemplar for the linguistic translation process.

**Methods:**

Using the World Health Organization guidelines, this work translated the ERIC compilation strategy names, short definitions, and thematic clusters through a three-step process: 1) forward translation into Spanish by a native Spanish-speaking implementation scientist, 2) back-translation into English by a bilingual global health researcher, and 3) piloting via virtual focus group discussions with bilingual researchers not conducting implementation research. To achieve a generalizable translation, recruitment targeted a multicultural group of Spanish-speaking researchers. At the conclusion of each step, the transdisciplinary research team (*N* = 7) met to discuss discrepancies and refine translations. The Spanish version of the ERIC compilation was finalized through group consensus. Reflections from research team meetings and focus group discussions were synthesized qualitatively.

**Results:**

Given that dialectical nuances exist between Spanish-speaking regions, efforts prioritized universally accepted terminology. Team discussions focused on difficult translations, word choice, and clarity of concepts. Seven researchers participated in two focus groups, where discussion surrounded clarity of concepts, alternative word choice for Spanish translations, linguistic formality, grammar, and conciseness. Translation difficulties highlighted lack of precision in implementation science terminology, and the lack of conceptual clarity of words underscored limitations in the application of the compilation.

**Conclusions:**

The work demonstrated the feasibility of translating implementation science resources. As one of the first systematic efforts to translate implementation resources, this study can serve as a model for additional efforts, including translation into other languages and the expansion to conceptual modifications. Further, this work yielded insights into the need to provide conceptual clarity in implementation science terminology. Importantly, the development of Spanish resources will increase access to conduct implementation research among Spanish-speaking populations.

**Supplementary Information:**

The online version contains supplementary material available at 10.1186/s43058-024-00616-6.

Contributions to the Literature:

- This research illustrates the feasibility of systematically translating implementation science resources through rigorous methods, thus serving as a model for future work.
- This effort provides a Spanish version of the Expert Recommendations for Implementing Change (ERIC) compilation.
- This work increases access to resources to conduct implementation science among Spanish-speaking populations.

## Background

Implementation science offers systematic approaches to increase the uptake of scientific discoveries [1]. The discipline has developed a robust repository of resources (e.g., theories, frameworks, and taxonomies) to facilitate the implementation of evidence-based practices. However, most of these resources exist only in English. Research has highlighted linguistic inaccessibility of implementation science resources as a barrier to the conduct of research in non-English speaking settings [2]. Further, the World Health Organization (WHO) outlined seven approaches to investing in implementation research in low- and middle-income countries (LMICs), including strengthening research capacity and assessing methods and frameworks for relevance to LMICs [3]. Language and culture are key factors to consider when evaluating the relevance of current resources. To facilitate implementation research and practice among non-English-speaking populations, particularly in LMICs, linguistic translation of resources is needed.

Translation of implementation science resources into Spanish could promote widespread impact. Spanish is the second most-spoken language in the world when counting native speakers and fifth most-spoken language when including non-native speakers [4]. Over 20 countries use Spanish as the official language, the majority of which are LMICs. Thus, translation into Spanish has the potential to reach a large proportion of the global population. Further, the literature suggests limited implementation research in Latin America, a predominately Spanish-speaking region [5–7]. For example, a search with keyword “implementation science” yielded 3,836 grants funded by the National Institutes of Health in fiscal year 2023, one common metric of implementation research, of which only 0.23% occurred in Latin America [8]. Due to the scarcity of implementation research among Spanish-speaking populations, particularly in Latin America, and the popularity of the language, a Spanish translation of resources could enhance research and practice to yield widespread public health impact in LMICs.

The WHO provides a gold standard approach for the translation and adaptation of instruments [9]. The guidelines outline a multi-step process including forward translation, back translation, and pre-testing with cognitive interviewing. Teams may have linguistically translated resources for individual implementation research or practice projects. However, few systematic translations that adhere to the WHO guidelines have occurred in the field of implementation science, and few gold standard translations are widely available to the research community (e.g., peer-reviewed publications) [10, 11]. Utilizing the WHO guidelines would help to maintain the integrity of implementation resources and increase the generalizability of translations.

This research sought to apply the WHO guidelines to systematically translate a widely used implementation science resource into Spanish as an exemplar for the crucial linguistic translation process: the Expert Recommendations for Implementing Change (ERIC) compilation. The ERIC compilation consists of 73 discrete implementation strategies [12]. Implementation strategies refer to the active methods used to increase the uptake of an evidence-based practice. Strategies reflect “how” implementation occurs, so they are critical for the success of the implementation process. The ERIC strategies were identified through literature review and extended and refined through a modified Delphi study and concept mapping exercise drawing upon the expertise of implementation scientists and practitioners [12–15]. They are organized into the following nine thematic clusters: use evaluative and iterative strategies, provide interactive assistance, adapt and tailor to context, develop stakeholder interrelationships, train and educate stakeholders, support clinicians, engage consumers, utilize financial strategies, and change infrastructure [15]. Given the disease-agnostic and comprehensive nature of the tool, the compilation has been widely used by researchers in diverse settings [16]. Further, while the ERIC strategies were developed and primarily used in the United States, researchers have applied the compilation to global settings [16], which suggests feasibility and value in translation. Published versions of the ERIC compilation currently only exist in English and German [11, 12], with a translation to Japanese in process. Thus, this study is timely with high potential for impact globally.

## Methods

This project was deemed exempt by the Institutional Review Board (IRB) at the University of Pennsylvania.

### Team structure

This research leveraged a transdisciplinary team to complete the translation process. Potential team members were purposively recruited via email based on relevant expertise (e.g., implementation science and WHO linguistic translation process). The final team consisted of seven researchers, including five implementation scientists, four global health researchers, and five Spanish speakers (*N* = 1 native speaker); individuals’ expertise was not exclusive to one category, and the team included three Spanish-speaking implementation science experts. One of the implementation science experts included the first author of the ERIC compilation [12]. All team members were appointed at academic institutions based in the United States at the time of the research. However, four team members have lived and/or collaborated in countries in Latin America (e.g., Guatemala, Ecuador, Nicaragua, and Dominican Republic) for extended periods of time, and one team member has extensive experience working with Spanish-speaking populations in the United States.

### Translation process

**Fig. 1:**
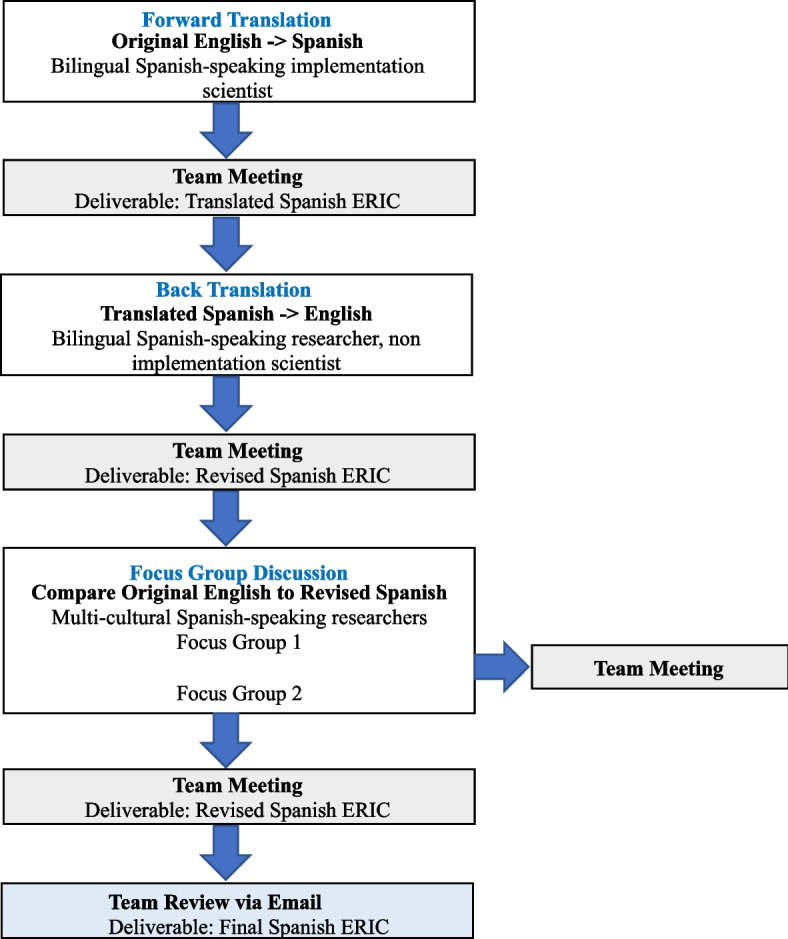
Overview of the translation process

The WHO guidelines for translation guided the research process. Briefly, translation followed a three-step process that involved forward translation (i.e., English to Spanish), back translation (i.e., Spanish to English), and piloting through focus group discussions (Fig. 1). The ERIC compilation content to translate included each implementation strategy name and short definition, which were organized by thematic cluster. The strategy numbers and cluster names from the original English compilation were used [13, 14]. Efforts focused on linguistic translation to achieve conceptual equivalence. Given that criterion equivalence or dialectical nuances exist between regions [17], the process aimed to select universally accepted terminology to increase relevance and applicability across cultures when possible (i.e., a word that would be understood by Spanish speakers in multiple settings rather than a word used predominately in one culture).

#### Forward translation

Forward translation focused on translating the original English version of the ERIC compilation into Spanish. This process was completed by a bilingual Spanish speaker who was born and academically trained in Guatemala with expertise in implementation science to increase familiarity with the content included in the compilation and, therefore, the accuracy of the translations. Forward translation began with a rapid review of the entire ERIC compilation in English to refresh familiarity with its structure, overarching concepts, terms, and thematic categories. The forward translator focused on translating the concept of the text rather than a word-by-word translation, striving for concise language. Concerted efforts to avoid local colloquialisms from Guatemala and Central America were made. When the forward translator was unsure of the Spanish term most used beyond the Central American region, multiple translations were provided. Google Translate was used to identify potential synonyms of such terms. Difficult-to-translate terms were highlighted for team discussion. A final review of the translated compilation occurred to ensure coherent use of terms and phrases throughout the document. Upon the conclusion of the preliminary translation, the translated document and translation log that identified difficulties were provided to the full research team. The research team met twice to review difficult concepts to translate and refine the documents before proceeding to the next step. This process resulted in a Spanish-translated version of the ERIC compilation. The forward translator received compensation for this work.

#### Back translation

Back translation aimed to translate the Spanish-translated version of the ERIC compilation into English. This process was completed by a bilingual Spanish speaker (Peruvian and Argentinian background) without expertise in implementation science to minimize familiarity with the content included in the compilation and, therefore, the accuracy of the translations. Back translation began by reading the entire Spanish-translated version of the ERIC compilation to identify any confusing phrases. The back translator proceeded with translating the document into English line by line. When the back translator was not familiar with a word (e.g., due to colloquialism in dialect), the term was searched in a Spanish dictionary. Difficult-to-translate terms (e.g., translations with more than one word or phrase, translations that suggested different meaning based on word choice, and translations that did not seem to flow with the rest of the English version) were documented in a log with alternative Spanish suggestions for team discussion. Once the entire document was translated into English, the back translator ensured that every instance of a repeated term was translated uniformly. If a repeated term was translated differently, both translations were noted in the log. The most appropriate and accurate translation in every instance was utilized for the preliminary translation draft. Upon the conclusion of the preliminary translation, the translated document and translation log that identified difficulties were provided to the full research team. The research team met once to review difficult concepts to translate and refine the documents before proceeding to the next step. This process resulted in a refined Spanish translation of the ERIC compilation. The back translator received compensation for this work.

#### Focus groups

Focus group discussions served to pilot the translations among target users. Potential participants were recruited via email, including distribution on relevant listservs (e.g., global health research affinity group and relevant university departments) and targeted outreach (i.e., research team contacted colleagues they knew worked with Spanish-speaking populations). Eligibility criteria included: 1) bilingual Spanish–English speaking, 2) conduct research with Spanish-speaking populations, and 3) do not conduct implementation research to minimize bias in translations due to familiarity with the compilation. Potential participants completed a brief interest form in REDCap that included demographic questions. Spanish proficiency was self-reported according to the following levels: a) native or bilingual, b) proficient/fluent, c) advanced, and d) intermediate and upper-intermediate (See Additional File 1 for definition of each category). All interest forms were reviewed by one member of the research team. Participants were excluded if they reported Spanish proficiency at the intermediate level or yes to the conduct of implementation research. If responses needed additional clarification (e.g., participant indicated “not sure” for the question about conducting implementation research), then a member of the research team contacted the individual for additional information via email. All eligible participants were invited for participation.

To increase access for global health colleagues, focus group discussions were facilitated virtually via Zoom. Participants were divided into two separate groups based on availability and the research team’s desire to diversify the backgrounds of participants in each discussion. The discussions began with a brief didactic presentation on implementation science and the goal of the focus group. Participants provided an overview of their current position and role, Spanish-speaking background, and experience working with Spanish-speaking populations. To facilitate review of the translations, both the original English and the refined Spanish translation documents were presented on the screen side-by-side. Participants concurrently reviewed the text line-by-line to provide feedback on needed edits or translations that did not make sense; they were encouraged to share all thoughts. With the permission of the participants, the focus group discussions were audio recorded, and detailed notes were documented. The research team met after the first focus group to review feedback and refine the approach for the second discussion through prioritization of words with multiple translation options and concepts with high confusion. Participants received a $50 gift card for participation.

### Analysis

Descriptive statistics were calculated for participant characteristics. Focus group discussions can produce a great deal of feedback, which may include differing opinions. A systematic process for determining which feedback to incorporate into the translated documents did not exist [11], so this step leveraged the transdisciplinary expertise of the research team. Feedback was categorized into two categories: 1) minor edits (e.g., grammar, punctuation, and concise language) with which the team agreed and applied directly, 2) substantive edits (e.g., alternative word choice, concepts discussed multiple times in team meetings, and points of confusion) for which the team discussed and applied with group consensus, and 3) exclusions (e.g., one-off opinions for alternative word choice) for which the team did not agree or apply. The research team met to review and agree upon edits to apply. The final document was reviewed by all research team members asynchronously. Reflections from both the research team meetings and the focus group discussions were synthesized qualitatively.

## Results

### Forward and back translation

The forward translation comprised both individual work and group discussion. The forward translator identified 12 items to discuss with the team. Additional File 2 provides an overview of the identified items. The team discussion surrounded word choice, particularly when a linguistic equivalent did not exist (e.g., lack of Spanish equivalent for “stakeholders” or for “shadowing” experts); clarity of concepts (e.g., use of Spanish word “primeros” to accurately capture early adopters); and grammar. The team made a concerted effort to discuss the integrity of the concepts in the compilation. For example, in implementation science, the concept “to tailor” reflects a specific action to modify the implementation strategy to fit the context. When translating this word into Spanish, potential translations included words that mean “to adapt” or “to adjust.” Given that adaptation often corresponds to the modification of an intervention for implementation, the team selected a translation that differentiated these concepts and captured the integrity of the original meaning of tailoring (“ajustar”). Documents were finalized for back translation, but the concept of “stakeholders” was highlighted for attention in future discussions for refinement.

Similarly, the back translation was completed through both individual work and group discussion. The back translator identified 13 items to discuss with the team. Additional File 3 provides an overview of the identified items. Overall, the back translator indicated that the Spanish translations were understandable despite a lack of knowledge of implementation science and required minor refinement. The back-translated document closely resembled the original English document. The team discussion focused on capturing the integrity of the implementation science concept in Spanish rather than the difficulty in translating the words (e.g., ensuring that the use of the word “clínicos” refers to clinicians and is not inclusive of clinics as well). Difficult concepts and phrases that emerged in both the forward translation and the back translation included: “stakeholders,” “knitting/weaving of social networks,” and the “utilize financing strategies” thematic cluster. The ancillary material was added for strategy 66 (use capitated payments) post hoc due to difficulty in translating the concept and the need for additional explanation.

### Focus groups

Seven individuals participated in the focus group discussions (*N* = 3 and 4) (Table 1). Gender distribution was approximately equal. Most participants identified as White (85.7%) and Hispanic or Latinx (85.7%). With regard to ethnicity, representation included: Argentine, Ecuadorian, Guatemalan, Mexican, Puerto Rican, and Venezuelan. The majority of participants self-reported Spanish language proficiency as native or bilingual (71.4%). Participants’ occupations varied, including researchers (faculty and staff), students, clinicians, and leadership (e.g., Executive Director of an international clinic). Most participants worked in a pediatric hospital (57.1%), followed by a university (28.6%) or multi-specialty outpatient clinic (14.3%). Combined, participants conducted research in 13 Spanish-speaking countries in Latin America, with the greatest proportion of researchers working in Colombia, Dominican Republic, and Peru (*N* = 3 for each country). One participant reported that he was not currently conducting research. Topically, research comprised diverse content areas (e.g., reproductive health, infectious diseases, medical imaging, oncology, eye disease, and social determinants of health).

**Table 1:** Focus group participant characteristics

Characteristic	N (%)
**Total**	7
**Gender**	
Man	3 (42.86)
Woman	4 (57.14)
**Position**	
Assistant professor	2 (28.57)
Master student	1 (14.29)
Research staff	2 (28.57)
Other^a^	2 (28.57)
**Institution Type**	
Multi-specialty outpatient clinic	1 (14.29)
Pediatric hospital	4 (57.14)
University	2 (28.57)
**Race**^**b**^	
American Indian or Alaskan Native	1 (14.29)
Asian	0
Black or African American	2 (28.57)
Native Hawaiian or Other Pacific Islander	0
White	6 (85.71)
**Ethnicity**	
Hispanic or Latinx	6 (85.71)
Argentine	1 (14.29)
Ecuadorian	1 (14.29)
Guatemalan	1 (14.29)
Mexican﻿	1 (14.29)
Puerto Rican	1 (14.29)
Venezuelan	1 (14.29)
**Spanish Language Proficiency**	
Advanced	2 (28.57)
Native or bilingual	5 (71.43)
**Research or Practice Region**^**c**^	
Argentina	2 (28.57)
Chile	1 (14.29)
Colombia	3 (42.86)
Costa Rica	1 (14.29)
Dominican Republic	3 (42.86)
Ecuador	1 (14.29)
El Salvador	1 (14.29)
Guatemala	2 (28.57)
Honduras	1 (14.29)
Mexico	1 (14.29)
Panama	2 (28.57)
Peru	3 (42.86)
United States	2 (28.57)
Not specified^d^	1 (14.29)

^a^Included clinician and clinic leadership roles

^b^Check all that apply; one participant did not report race

^c^List all that apply

^d^Participant not currently conducting research. Region not specified

The first focus group lasted three hours, including a break of 10 minutes, and the second focus group lasted two hours excluding a mid-point break due to scheduling constraints. Overall, the focus groups provided valuable insight for refining the translations. Discussion surrounded clarity of concepts (i.e., not understanding the implementation science specific language or “jargon”), alternative word choice for Spanish translations (i.e., not how participants would translate into Spanish; acknowledging bias from Spanish-speaking background), formality of the language (e.g., participants thought some translations read casually), grammar (e.g., use of verb tenses), and conciseness of translations (e.g., deletion of unnecessary prepositions).

### Spanish version of ERIC

Table 2 provides the final translated version of the ERIC compilation and accompanying short definitions.

**Table 2:** Spanish translation of ERIC

Taxonomía de las Recomendaciones de Expertos para Implementar Cambio (ERIC por sus siglas en inglés)	
Estrategia de implementación	**Definición**
**Utilizar estrategias iterativas de evaluación**	
4. Evaluar la disposición e identificar barreras y facilitadores	Evaluar varios aspectos de una organización para determinar su grado de disposición para implementar, las barreras que podrían impedir la implementación y las fortalezas que se pueden aprovechar en el proceso de implementación
5. Auditar y proveer retroalimentación	Recopilar y resumir información sobre desempeño clínico durante un período de tiempo específico y compartírselos a los médicos y administradores para monitorear, evaluar y modificar el comportamiento del proveedor
56. Reexaminar deliberadamente la implementación	Monitorear el progreso y ajustar las prácticas clínicas y las estrategias de implementación para mejorar continuamente la calidad de la atención
26. Desarrollar e implementar herramientas para el monitoreo de la calidad	Desarrollar, probar e introducir en los sistemas de monitoreo de calidad la información: el lenguaje, protocolos, algoritmos, estándares y medidas apropiadas (de procesos, resultados del paciente/consumidor y resultados de implementación) que comúnmente son específicos de la innovación que se está implementando
27. Desarrollar y organizar sistemas de monitoreo de calidad	Desarrollar y organizar sistemas y procedimientos de monitoreo de los procesos clínicos y/o los resultados con el fin de garantizar y mejorar la calidad
23. Desarrollar un plan de implementación formal	Desarrollar un plan de implementación formal que incluya todas las metas y estrategias. El plan debe incluir: 1) objetivo/propósito de la implementación; 2) alcance del cambio (ej., qué unidades organizativas se ven afectadas); 3) cronograma e hitos; y 4) medidas apropiadas de desempeño/progreso. Utilice y actualice este plan para guiar el proceso de la implementación a lo largo del tiempo
18. Realizar una evaluación de las necesidades locales	Recolectar y analizar datos relacionados con las necesidades de la innovación
61. Escalar la implementación por etapas	Dividir la implementación en etapas iniciando con pequeños pilotos o proyectos de demostración y pasando gradualmente a una implementación en todo el sistema
46. ​​Obtener y utilizar los comentarios de los pacientes/consumidores y familiares	Desarrollar estrategias para facilitar la retroalimentación de pacientes/consumidores y familiares sobre el proceso de la implementación
14. Realizar pequeñas pruebas de cambio cíclicamente	Implementar cambios por ciclos utilizando pequeñas pruebas de cambio antes de realizar cambios en todo el sistema. Es beneficioso realizar mediciones sistemáticas de las pruebas de cambio y analizar los resultados para entender cómo hacerlo mejor. Este proceso continúa repetidamente a lo largo del tiempo, refinando en cada ciclo
**Proveer asistencia interactiva**	
33. Facilitación	Un proceso interactivo de resolución de problemas y apoyo que ocurre en un contexto en la que se ha identificado la necesidad de mejorar y una relación interpersonal de apoyo
54. Proveer asistencia técnica local	Desarrollar y utilizar un sistema para proveer asistencia técnica enfocada en retos de implementación utilizando personal local
53. Proveer supervisión clínica	Proveer supervisión continúa con foco en la innovación a los clínicos. Proveer capacitación a los supervisores clínicos que supervisarán a los clínicos que proveen la innovación
8. Centralizar la asistencia técnica	Desarrollar y utilizar un sistema centralizado para proveer asistencia técnica enfocada en retos de implementación
**Adaptar y ajustar al contexto**	
63. Ajustar las estrategias	Ajustar las estrategias de implementación para superar las barreras y aprovechar los facilitadores que se identificaron en una previa recopilación de datos
51. Promover la adaptabilidad (de la innovación)	Identificar formas en las que una innovación clínica puede adaptarse para cumplir con las necesidades locales, aclarando qué elementos de la innovación deben mantenerse para preservar la fidelidad
67. Usar expertos en datos	Involucrar, contratar y/o consultar con expertos para guiar a la gerencia sobre el uso de los datos generados a través del proceso de implementación
68. Utilizar técnicas de almacenamiento de datos	Integrar registros clínicos en las instalaciones y organizaciones para facilitar los procesos de implementación
**Desarrollar interrelaciones entre las partes interesadas**	
35. Identificar y preparar campeones	Identificar y preparar a personas que se dedican a apoyar, comercializar e impulsar una implementación, superando la indiferencia o resistencia que la intervención podría provocar en una organización
48. Organizar reuniones del equipo clínico de implementación	Desarrollar y apoyar a los equipos clínicos que están implementando la innovación y darles tiempo protegido para reflexionar sobre la implementación, compartir lecciones aprendidas y apoyarse mutuamente a aprender
57. Reclutar, designar y capacitar líderes	Reclutar, designar y capacitar personas para liderar el cambio
38. Informar a los líderes de opinión locales	Informar a los proveedores que son reconocidos por sus colegas como líderes de opinión o personas que influyen en la educación sobre la innovación clínica esperando que estos influyan a sus colegas adopten la innovación
6. Formar una coalición	Reclutar y cultivar relaciones con colaboradores que forman parte del proceso de implementación
47. Obtener compromisos formales	Obtener compromisos escritos de los colaboradores claves que describan lo que harán para implementar la innovación
36. Identificar a los primeros adoptadores (de la innovación)	Identificar a los primeros adoptadores de la innovación en el sitio local para aprender de sus experiencias utilizando la innovación
17. Realizar discusiones para alcanzar consensos	Involucrar a proveedores locales y otras partes interesadas en discusiones acerca de si el problema es importante y si la innovación clínica es apropiada para resolverlo
7. Recolectar y compartir el conocimiento	Recolectar información en sitios locales sobre cómo los implementadores y clínicos lograron que algo funcionara en su sitio, y luego compartir con otros sitios
64. Utilizar consejos consultivos y grupos de trabajo	Crear e involucrar a un grupo formal de partes interesadas que representen múltiples tipos de intereses para proveer retroalimentación y consejos sobre la implementación y obtener recomendaciones para mejorarla
65. Usar un consultor de implementación	Consultar con expertos en implementación
45. Modelar y simular el cambio	Modelar o simular el cambio que se implementará antes de iniciar la implementación
72. Visitar otros sitios	Visitar sitios que hayan implementado algo similar de forma exitosa
40. Involucrar a juntas directivas	Involucrar a las estructuras de liderazgo existentes (ej. juntas directivas, juntas de personal médico) en la implementación, incluyendo en la revisión de información sobre el proceso de implementación
25. Desarrollar un glosario de implementación	Desarrollar y distribuir una lista de términos que describa la innovación, la implementación y las partes interesadas en el cambio organizacional
24. Desarrollar colaboraciones académicas	Colaborar con una universidad o unidad académica con el fin de cooperar en las capacitaciones e incorporar habilidades de investigación a un proyecto de implementación
52. Conectar redes sociales	Identificar y aprovechar las relaciones y redes de trabajo sociales de alta calidad disponibles dentro y fuera de la organización, las unidades organizativas, los equipos, etc. para promover el intercambio de información, la resolución colaborativa de problemas y un/a objetivo/ visión común relacionado/a con la implementación de la innovación
**Capacitar y educar a las partes interesadas**	
19. Realizar capacitaciones continuamente	Planificar y realizar capacitaciones de forma continua sobre la innovación clínica
55. Proveer consultas continuamente	Brindar consultas continuamente con uno o más expertos enfocados en las estrategias que facilitan la implementación de la innovación
29. Desarrollar materiales educativos	Desarrollar y diseñar manuales, conjuntos de herramientas y otros materiales de apoyo para facilitar que las partes interesadas aprendan sobre la innovación y para que los clínicos aprendan cómo proveer la innovación clínica
23. Realizar capacitación dinámica	Usar varios métodos de enseñanza adaptándose a los distintos estilos de aprendizaje en los contextos de trabajo y diseñar una capacitación interactiva sobre la innovación
31. Distribuir materiales educativos	Distribuir materiales educativos (incluyendo guías, manuales y conjuntos de herramientas) en persona, por correo y/o electrónicamente
71. Utilizar estrategias de capacitación de capacitadores	Capacitar a los clínicos u organizaciones designados para capacitar a otros en la innovación clínica
15. Efectuar reuniones educativas	Organizar reuniones dirigidas a diferentes partes interesadas (ej., proveedores, administradores, otras partes interesadas de la organización y partes interesadas de la comunidad, pacientes/consumidores y familias) para enseñarles sobre la innovación clínica
16. Realizar visitas de extensión educativa	Hacer que una persona capacitada se reúna con los proveedores en sus ámbitos de trabajo para educarlos acerca de la innovación clínica con la intención de cambiar la práctica de los proveedores
20.Crear un equipo de aprendizaje	Facilitar la formación de grupos de proveedores u organizaciones de proveedores y fomentar ámbitos de aprendizaje colaborativo para mejorar la implementación de la innovación clínica
60. Observar a expertos	Facilitar que personas claves observen directamente a expertas involucrándose en la práctica que se busca cambiar o innovar
73. Trabajar con instituciones educativas	Promover que instituciones educativas capaciten a los clínicos en la innovación
**Apoyar a clínicos**	
32. Facilitar la transferencia de datos clínicos a los proveedores	Proporcionar datos lo más cercano posible a tiempo real sobre mediciones claves de proceso / resultados utilizando formas / canales de comunicación integrados de forma que promuevan el uso de la innovación
58. Enviar recordatorios a los clínicos	Desarrollar sistemas de recordatorios para ayudar a los clínicos a recordar información y/o motivarlos a usar la innovación clínica
30. Desarrollar acuerdos de uso de recursos compartidos	Desarrollar colaboraciones con organizaciones que tengan los recursos necesarios para implementar la innovación
59. Revisar los roles profesionales	Cambiar y revisar los roles entre profesionales que proveen atención y rediseñar las características de trabajo
21. Crear nuevos equipos clínicos	Cambiar quién forma parte del equipo clínico, agregando diferentes disciplinas y diferentes habilidades para aumentar la posibilidad de que la innovación clínica se provea (o que sea provea con más éxito)
**Involucrar a los usuarios**	
41. Involucrar a los pacientes/usuarios y familiares	Involucrar o incluir a los pacientes/usuarios y familias en el proceso de implementación
39. Intervenir en los pacientes/usuarios para mejorar el uso y la adherencia	Desarrollar estrategias con pacientes para fomentar y resolver problemas relacionados con la adherencia
50. Apoyar a los pacientes/usuarios para que sean participantes activos	Apoyar a los pacientes/usuarios para que participen activamente en su atención, hacer preguntas y específicamente para preguntar sobre las guías de atención, la evidencia que soporta las decisiones clínicas o sobre la disponibilidad de tratamientos basados en evidencia
37. Aumentar la demanda	Intentar influir en el mercado de la innovación clínica para aumentar la intensidad de la competencia y aumentar la madurez del mercado de la innovación clínica
69. Utilizar medios de comunicación masivos	Utilizar medios de comunicación para alcanzar a un gran número de personas y difundir la innovación clínica
**Utilizar estrategias financieras**	
34. Financiar y contratar para favorecer la innovación clínica	Los gobiernos y otros pagadores de servicios emiten solicitudes de propuestas para ofrecer la innovación, utilizan procesos de contratación para motivar a los proveedores a ofrecer la innovación clínica y desarrollan nuevas fórmulas de financiación que hacen más probable que los proveedores entregarán la innovación
1. Obtener nuevas fuentes de financiamiento	Acceder a fuentes de financiamiento, nuevos o existentes, para facilitar la implementación
49. Incluir la innovación en listados/formularios de tarifas por servicios prestados	Incluir la innovación clínica en listados de actividades por las que los proveedores pueden ser reembolsados (ej. Incluir un medicamento en un formulario, hacer que un procedimiento sea reembolsable)
2. Modificar los sistemas de incentivos / remuneraciones	Incentivar la adopción e implementación de la innovación clínica
42. Facilitar el proceso de facturación	Hacer que el proceso de facturar la innovación clínica sea más fácil
3. Modificar las tarifas de los pacientes/usuarios	Crear sistemas de tarifas en las que los pacientes/usuarios paguen menos por los tratamientos priorizados (la innovación clínica) y más por los tratamientos menos priorizados
70. Usar otros sistemas de pago	Introducir estrategias de pago (en una categoría general)
28. Desarrollar desincentivos	Proveer desincentivos financieros por no implementar o utilizar las innovaciones clínicas
66. Usar pagos fijos^a^	Pagar a los proveedores o sistemas de atención una cantidad fija por paciente/usuario por proveer atención clínica
**Cambiar la infraestructura**	
44. Imponer el cambio	Que el liderazgo declare que la innovación es una prioridad y que está determinado a implementarla
12. Cambiar los sistemas de registro	Cambiar los sistemas de registro para facilitar una mejor evaluación de la implementación o de resultados clínicos
11. Cambiar la estructura física y el equipo	Evaluar la configuración actual y adaptar la estructura física y/o el equipo (ej., cambiar el diseño de una habitación, agregar equipo) según sea necesario, para acomodarse mejor a la innovación de interés
22. Crear o cambiar los estándares de acreditación y/o licencia	Crear una organización que certifique a los clínicos que participan en la innovación o alentar a una organización existente a hacerlo. Cambiar los requisitos gubernamentales de certificación o licencia profesional para incluir la provisión de la innovación. Modificar los requisitos de educación continua para alinear la práctica profesional con la innovación
13. Cambiar los sitios de servicio	Cambie la ubicación de los sitios de servicio clínico para aumentar el acceso a ellos
9. Cambiar los requisitos de acreditación o membresías	Esforzarse por alterar los estándares de acreditación para que requieran o promuevan el uso de la innovación clínica. Modificar los requisitos necesarios para ser miembro de una organización con el fin de que se exija o aliente a utilizar la innovación clínica a los que quieran afiliarse con la organización
62. Comenzar una organización de difusión	Identificar o iniciar una nueva organización responsable de difundir la innovación clínica. Puede ser una organización con o sin fines de lucro
10. Cambiar las leyes de responsabilidad civil	Participar en procesos de implementación para reformar las leyes de responsabilidad civil para hacer que los clínicos estén más dispuestos a ofrecer la innovación clínica

^a^Material Suplementario: Esta es una estrategia de implementación en la medida que libera al clínico para proveer servicios que antes podría haber estado desincentivado a proveer en una estructura de “pagos / honorarios por servicios prestados.” Esto puede ser útil para motivar a los clínicos a utilizar ciertas innovaciones clínicas. Frecuentemente, estos cambios son parte de cambios de políticas que alteran las estructuras de pagos, alteran la cobertura, o agregan artículos a formularios de reembolso

### Key themes from the translation process

Translation difficulties highlighted issues related to the precision of language in the field of implementation science. For example, all individuals, including the research team comprised of expert implementation scientists, expressed difficulty in translating abstract concepts (e.g., “promote network weaving,” “educationally influential” in the definition to inform local opinion leaders, and distinction to “identify early adopters” as first and/or early adopters as aligned with theory). The need to engage in in-depth discussion to understand the meaning of the original term in English challenges the usability of implementation science resources (i.e., how user-friendly tools are). In addition to abstract concepts, focus group participants (i.e., non-implementation scientists) described some terms in English as “confusing” (e.g., local opinion leaders, funding formulas, and complexity index).

In addition, the discussion underscored limitations in the application of the ERIC compilation. Developed in the United States, the ERIC compilation consists of concepts relevant to the United States context. Throughout the translation process, difficulties in translation occurred due to irrelevant concepts in Spanish-speaking settings. For example, some of the strategies included in the thematic cluster “utilize financial strategies” describe components of the healthcare systems in the United States, which do not apply to settings in Latin America. Multiple factors may contribute to the lack of relevance, such as the difference in the structure of payment systems between countries (e.g., use of capitated payments). The focus group and research team discussions emphasized this point through in-depth discussion of confusion surrounding health insurance concepts included in the ERIC compilation. In addition to financial strategies, discussion focused on behavioral strategies as well. For instance, practices such as “shadowing experts” do not commonly occur in Latin American countries.

## Discussion

This work systematically translated the ERIC compilation into Spanish, a widely spoken language worldwide, to serve as an exemplar of gold-standard linguistic translation. This process provides a new tool for the implementation science community, which can empower more locally led research among Spanish-speaking populations. Further, this process yielded valuable insights into the limitations of the current implementation science resources and future directions.

This study contributes to efforts to linguistically translate implementation science resources [10, 11, 18], a priority for the field [5]. The use of the WHO guidelines for translation helped to maintain the integrity of the concepts in the ERIC compilation. Further, the participation of individuals with diverse Spanish-speaking backgrounds facilitated a more universal translation, which increased the generalizability of the Spanish version of the compilation. Future translation efforts should follow this gold standard process. Specifically, successful translation suggests the feasibility of translating other implementation science resources (e.g., Consolidated Framework for Implementation Research [19] into Spanish. Further, this work suggests feasibility for applying these rigorous methodologies to translate the ERIC compilation into other languages. Thus, this study provides a model for multiple translation efforts. In future translations, however, the expansion of the process to also include a focus group of Spanish-speaking implementation scientists may help to overcome difficulties in translation and strengthen the translation process.

In addition, this study can contribute to more locally led research and practice among non-English-speaking populations. Translation grows the implementation science toolkit by overcoming linguistic barriers and producing more accessible resources for individuals. Increased accessibility reduces the reliance on partnering with English-speaking colleagues. The development of a Spanish version of the ERIC compilation can help empower constituents to lead implementation research and practice among Spanish-speaking populations, particularly in Latin America. However, it is important to note the limitation of the US-centric nature of the ERIC compilation and the potential need for cultural adaptations of the resource to increase appropriateness. Further, it is important to note that the provision of a translated compilation alone will not increase the conduct of implementation research in the absence of capacity building in implementation science. The focus groups engaged participants who conduct research among Spanish-speaking populations but do not conduct implementation research. The primary purpose of this recruitment was to minimize the potential for bias due to familiarity with concepts, as aligned with guidelines for translation. However, the participation of such individuals introduces global health researchers to a new discipline for potential integration into their respective research, which extends the impact of this work.

As the field continues to develop new implementation science resources, attention to the limitations of the resources highlighted through these translation efforts is critical. For example, to overcome the challenges with the precision of implementation science language, initial development of implementation science resources could involve translation of the resource into multiple languages or review of the resource by multi-lingual individuals to proactively identify confusing terms for revision. In addition, development of implementation science resources could include non-researchers for increased conceptual clarity. To address the limitations related to the relevance of concepts for use in countries other than the United States, future directions could adapt implementation science resources through systematic conceptual translations (e.g., replace ERIC compilation strategies with strategies that are relevant for each context; see Kirchner et al., 2023 for a suggested process of adapting ERIC strategies for other contexts [20]. Further, development of implementation science resources should include constituents in LMICs at the onset [5].

This research had limitations. First, the Spanish-speaking backgrounds of the research team and participants may have influenced the translations. The Spanish language has many nuances based on regional dialects [17], so translations reflect the experience and knowledge of the group. To mitigate these influences and produce a generalizable translation, the team recruited a multicultural group of individuals and aimed to select universally accepted words. However, the translation may be more relevant for countries in Latin America given the Spanish-speaking backgrounds of participating individuals and lack of inclusion of a participant from Spain. Future efforts could incorporate additional translations relevant to specific Spanish-speaking regions or explanations of differences in application based on cultural contexts. Second, a standardized process for deciding which feedback to incorporate into the final translated deliverables did not exist. Approaches utilized expert consensus among the transdisciplinary, diverse research team. Third, the virtual focus group discussions may have impacted participant engagement (e.g., distractions during discussions). However, this process increased access to global health colleagues and enabled an easier display of materials for review and, potentially, an easier contribution for some participants. Fourth, focus group discussions may have experienced bias due to participants’ hesitancy to critique translations. The team mitigated this potential issue by emphasizing that honest feedback would strengthen the work. Explicit discussion at the beginning of the focus groups corrected the issue and resulted in more robust feedback.

## Conclusions

This process developed a Spanish version of the ERIC compilation. Successful translation demonstrated feasibility in the systematic translation of existing implementation science resources, which can serve as a model for future studies involving translation into other languages. The development of resources for Spanish-speaking individuals will increase access to conduct implementation research in Spanish-speaking countries to address the scarcity of research in this context. Efforts should continue to systematically translate implementation science resources, both into Spanish and other languages common in LMICs, as well as explore the expansion of focus to conceptual modifications to develop resources for non-academic constituents and cultural modifications for global health partners.

### Supplementary Information

Supplementary Material 1.

## Data Availability

Materials are available upon reasonable request to the corresponding author.

## Abbreviations

ERIC: Expert Recommendations for Implementing Change
LMICs: Low- and middle-income countries
WHO: World Health Organization

## References

[1] Lane-Fall MB, Curran GM, Beidas RS (2019). Scoping implementation science for the beginner: locating yourself on the "subway line" of translational research. BMC Med Res Methodol.

[2] Schultes MT, Finsterwald M, Brunkert T, Kien C, Pfadenhauer L, Albers B (2022). Barriers and facilitators for conducting implementation science in German-speaking countries: findings from the Promote ImpSci Interview Study. Glob Implement Res Appl.

[3] World Health Organization. Seven approaches to investing in implementaiton research in low- and middle-income countries. Available from: https://tdr.who.int/publications/m/item/2020-09-29-seven-approaches-to-investing-in-implementation-research-in-low-and-middle-income-countries

[4] Eberhard DM, David M, Simons GF, Fennig CD (2022). Ethnologue: languages of the world. (25th ed.).

[5] Van Pelt AE, Beidas RS, Baumann AA, Castillo-Neyra R (2023). Recommendations for empowering partners to conduct implementation research in Latin America to advance global health. Glob Implement Res Appl.

[6] Kirk MA, Kelley C, Yankey N, Birken SA, Abadie B, Damschroder L (2016). A systematic review of the use of the Consolidated Framework for Implementation Research. Implement Sci.

[7] Ojo T, Kabasele L, Boyd B, Enechukwu S, Ryan N, Gyamfi J (2021). The role of implementation science in advancing resource generation for health interventions in low- and middle-income countries. Health Serv Insights.

[8] National Institutes of Health Health. RePORTER. Available from: https://reporter.nih.gov/search/g8olWagh40yKPn70_r-a9g/projects?fy=2023

[9] World Health Organization. Process of translation and adaptation of instruments. Available from: http://www.who.int/substance_abuse/research_tools/translation/en/.

[10] Baumann AA, Vazquez AL, Macchione AC, Lima A, Coelho AF, Juras M (2022). Translation and validation of the evidence-based practice attitude scale (EBPAS-15) to Brazilian Portuguese: examining providers' perspective about evidence-based parent intervention. Child Youth Serv Rev..

[11] Regauer V, Seckler E, Campbell C, Phillips A, Rotter T, Bauer P (2021). German translation and pre-testing of Consolidated Framework for Implementation Research (CFIR) and Expert Recommendations for Implementing Change (ERIC). Implement Sci Commun.

[12] Powell BJ, Waltz TJ, Chinman MJ, Damschroder LJ, Smith JL, Matthieu MM, Proctor EK, Kirchner JE (2015). A refined compilation of implementation strategies: results from the Expert Recommendations for Implementing Change (ERIC) project. Implement Sci..

[13] Powell BJ, McMillen JC, Proctor EK, Carpenter CR, Griffey RT, Bunger AC (2012). A compilation of strategies for implementing clinical innovations in health and mental health. Med Care Res Rev.

[14] Waltz TJ, Powell BJ, Chinman MJ, Smith JL, Matthieu MM, Proctor EK (2014). Expert Recommendations for Implementing Change (ERIC): protocol for a mixed methods study. Implement Sci.

[15] Waltz TJ, Powell BJ, Matthieu MM, Damschroder LJ, Chinman MJ, Smith JL (2015). Use of concept mapping to characterize relationships among implementation strategies and assess their feasibility and importance: results from the Expert Recommendations for Implementing Change (ERIC) study. Implement Sci.

[16] Lovero KL, Kemp CG, Wagenaar BH, Giusto A, Greene MC, Powell BJ (2023). Application of the Expert Recommendations for Implementing Change (ERIC) compilation of strategies to health intervention implementation in low- and middle-income countries: A systematic review. Implement Sci.

[17] Hualde JI, Olarrea A, O'Rourke E (2012). The Handbook of Hispanic Linguistics.

[18] Padovez MC, Inguane C, Luz RA, Juskevicius LF. Quadro Conceitual Consolidado para Pesquisa de Implementação - CFIR. Available from: https://cfirguide.org/wp-content/uploads/2021/02/CFIR-Portuguese-v1.0-20201230_revMC.pdf.

[19] Damschroder LJ, Reardon CM, Widerquist MAO, Lowery J (2022). The updated Consolidated Framework for Implementation Research based on user feedback. Implement Sci..

[20] Kirchner JE, Waltz TJ, Powell BJ, Woodward EN, Smith JL, Proctor EK, Brownson R, Colditz GA, Proctor E (2023). Implementation Strategies. Dissemination and implementation research in health: translating science to practice.

