# Supplementary material for: Spanish translation of the Expert Recommendations for Implementing Change (ERIC) compilation
Source: Implement Sci Commun. 2024 Jul 17;5:77. doi: 10.1186/s43058-024-00616-6 (PMC11253493; doi:10.1186/s43058-024-00616-6)
Supplement: Supplementary file 1 — Supplementary Material 1. [file 43058_2024_616_MOESM1_ESM.docx]

**Additional Files**

**Additional File 1. Definitions for Self-Reported Spanish-Speaking Skill Level**

| **Language Level** | **Definition** |
| --- | --- |
| Native or bilingual | Full mastery of the language through either upbringing or advanced education. |
| Proficient/fluent | This means full professional working proficiency. You have fluid speech and master reading and writing, but with a less advanced vocabulary than a native speaker. The proficient versus fluent are a matter of grammar and colloquialisms. |
| Advanced | You are skilled enough to carry complex conversations but still put in the conscious effort when speaking and writing. |
| Intermediate and upper-intermediate | You can carry out basic conversations in a wide variety of situations, but you still make grammar mistakes. You have limited working proficiency. |

**Additional File 2. Difficult Translations from Forward Translation.**

| TABLE 1  Difficult Translations in Forward Translation | | | | |
| --- | --- | --- | --- | --- |
| **ERIC Strategy^a^** | **English Phrase** | **Forward Translation** | **Team Notes** | **Final Translation** |
| 4 | implementation effort | iniciativa de implementación | **Forward translator:** The literal translation would be "esfuerzo de implementación" but it does not sound right. I am using iniciativa de implementación, although this is not 100% accurate as it translates back as "Implementation initiative."  **Team:**   - Implementation effort feels like it’s capturing the idea of the process/attempt of implementing the thing. - Team agrees that “esfuerzo” feels more like the effort made by an individual (e.g., physical or mental effort to do something.) - Alternative potential suggestion: “intento (attempt) de implementación” - Initiative feels like it’s more organized than attempt. - Some of the team interprets implementation effort as how much work it takes to actually do it. - The word “process” seems to capture the construct best- proceso de implementación | proceso de implementación |
| 26 | inputs | entradas | **Forward translator:** there's no Spanish equivalent to "input.” The closest word is "entrada."  **Team:**   - “Entradas” could suggest input related to computing. - Alternative potential suggestions that may be oversimplified: “información” (information), “datos” (data) - Entrada feels too specific. - Information seems fine to capture. - There is no word for this in Spanish. | la información |
| Adapt and tailor to context cluster | tailor | ajustar | **Forward translator:** Tailor can be translated into “ajustar” or “adaptar” in this context. I am using tailor as this seems more granular than "adaptar," which translates back as "adaptation."  **Team:**   - The team is conflicted on this translation as well. - “Adaptar” fits well. However, the larger bucket for this strategy is to “adapt and tailor.” Adaptar works well for adapt, so we agree that we can come up with a more specific word for tailor to reflect the changes made to meet a contextual need. - “Ajustar” means adjust, but it’s used more for physical things. - “Adaptar” translates back to adapt. - The concept means not one size fits all- tailor to this individual concept. - Alternative potential suggestion: “ajustar a individualizer”? - Concerns that we could be tailoring to organization and not an individual. | ajustar |
| 17 | stakeholders | personas interesadas (stakeholders) | **Forward translator:** There's no great translation of stakeholders in Spanish. Most documents use "partes interesadas," but this seems confusing in this context. I’m using "personas interesadas" and leaving (stakeholders) in parenthesis, since this is an English term that is frequently used in Latin America, particularly within non-profit organizations.  **Team:**   - The team likes “personas” (persons) more than “partes” (think of part) because we feel like we are referring to the people in implementation science. - If you’ve seen “stakeholder” commonly used in Latin America, then it makes sense to include-especially to make sure we’re capturing the intended meaning. - Sometimes folks use “stakeholders” in Latin America. - If there is already a word to mean stakeholder, then we should use that word. - Stakeholders can be at different levels not just patient-level. - Some of the team prefers “partes.” - Note that “stakeholders” is a less preferred term now in English, so we may not want to use this word. - Could we say partners? - Alternative potential suggestion: “colaboradores interesadas” – collaborators - Are all stakeholders collaborators? - “Partes interesadas” is used by non-profits. | personas interesadas |
| 38 | educationally influential | influyen en la educación | **Forward translator:** The literal translation would be "educativamente influyentes," but this sounds very weird.  **Team:**   - This term is referring to providers, but some of the team is not clear on what “educationally influential” even means. - Alternative potential suggestion: “personas influyente” (influential persons)- although that loses the educational piece. - Some of the team suggests dropping the “educational influente” because we are referencing the person. - Alternative potential suggestion: “personas que influyen en educación” | personas que influyen en la educación |
| 36 | early adopters | primeros adoptadores | **Forward translator:** "Primeros adaptadores" literally translates to "first adopters," which is not the same as "early adopters." Early adopters translates to "adoptadores tempranos," but this does not sound right in Spanish.  **Team:**   - Some of the team agrees that early adopters are not necessarily the first to adopt. They’re fall in the second group of people who adopt a thing, which is still before the majority of people. We think we should change the word “primero” to avoid falsely representing the innovators, unless it’s clear that “innovadores” represents the first group of people. - Alternative potential suggestion: “anticipados” - No one would say “tempranos” in Spanish. - We wouldn't really use the word “anticipados” to refer to people in Spanish. - The English ERIC term is meant to capture both innovators and early adopters, so we can capture both in the Spanish translation. | primeros adoptadores |
| 52 | network | redes sociales | **Forward translator:** The word "network" translates to "red" in Spanish, which can mean many things. I added "redes sociales" to make it more explicit. We can change the word to "redes" if we prefer a translation closer to the literal meaning in English.  **Team:**   - Some of the team thinks of computers when I see the word “red.” - We agree with the addition of “social” to specify that it’s a social network and referring to a group of people. | redes sociales |
| 60 | shadow other experts | observar a expertos | **Forward translator:** There's no term for "professional shadowing" or similar in Spanish. The closest word is "observar" or "observar la practica de expertos."  **Team:**   - Observing is not a common practice in Latin America. - Observar feels like it’s capturing the integrity of the concept in English. | observar a expertos |
| Utilize financial strategies cluster | Utilize financial strategies | utilizar estrategias finacieras | **Forward translator:** This section was extremely challenging to translate to Spanish. Many of these terms are related to US-based insurance companies, so it's hard to translate to something with real meaning, at least in most Latin American countries.  **Team:**   - The current translation works well for a literal translation, but there are conceptual concerns. - Latin America does not have the same health insurance systems as the United States, which makes the translation of these strategies difficult. - Should we be translating the concepts to the context in Latin America? However, this is a different focus of the work, and there are multiple health systems in Latin American countries (i.e., different context per translation). - As the translation stands, is would it generalize to other countries? - This is a critique of the original ERIC compilation that it is US-centric. - There are Spanish-speaking populations in the US, Spain, etc. so lack of relevance for LAC may not be the only factor to consider. For example, Spanish-speaking populations in the US could use these strategies. | utilizar estrategias finacieras |
| 2 | Work to incentivize the adoption and implementation of the clinical innovation. | Trabajar para incentivar la adopción e implementación de la innovación clínica. | **Forward translator:** This phrase seems incomplete, as it does not fully relate to the implementation strategy name.  **Team:**   - The idea of incentivizing is related to the thematic idea of using financial strategies, because incentives are often payment or financial in nature. - We read this definition as put effort toward changing (alter in strategy name) the structure for incentives or allowances. - The team agrees that the original phrase is confusing. - Alternative potential suggestion: “tratar” (try to) instead of “trabajar” - Do we need to include the “work to” part of the phrase? Can we just say to incentivize? - Forward translator is not familiar with the allowance structure (i.e., additional compensation). | Incentivar la adopción e implementación de la innovación clínica. |
| 70 | Introduce payment approaches (in a catch-all category). | Introducir tipos de pago (en una categoría general). | **Forward translator:** I am not sure what this means in English.  **Team:**   - The team thinks it refers to different strategies for paying for clinical services. - Alternative potential suggestion: “estrategias de pago” (payment strategies/approaches) - Would it be accurate to refer to compensation strategies? | Introducir estrategias de pago (en una categoría general). |
| 10 | Change liability laws | Cambiar las leyes de responsabilidad civil | **Forward translator:** Although I am not familiar with legal terms, I found that in other documents the term "liability laws" was translated into "leyes de responsabilidad civil" which is why I am using this term. However, feel free to remove "civil" if this does not seem to reflect the original meaning.  **Team:**   - Some of the team thinks that liability is simply referring to the responsibility (of the clinicians in this case). They are unclear on whether or not the word “civil” changes the meaning. - “Civil” still captures the integrity of the concept. - Liability laws are just getting started in Latin America. | cambiar las leyes de responsabilidad civil |

**^a^**The phrase may appear in multiple ERIC strategies, so reference is not exhaustive of all occurrences.

**Additional File 3. Difficult Translations from Back Translation.**

| TABLE 2  Difficult Translations in Back Translation | | | | |
| --- | --- | --- | --- | --- |
| **ERIC Strategy^a^** | **Spanish Phrase** | **Back Translation** | **Team Notes** | **Final Translation** |
| 27 | monitoreo de calidad | monitor quality | **Back translator:** Is this supposed to be quality control? (Control de calidad)  **Team:**   - Written as “quality monitoring systems” in the original- Conceptually it does feel like quality control, although the language was different. - Does quality control = quality monitoring? - Note from back translation is there because there is an English phrase that’s more familiar to her. It is not really a problem with the translation. - Quality control feels like manufacturing; more heavy-handed than it is intended to be. - Some team members like shift monitoring- likes monitoring more than control. - The connotation feels different between the two- control is more paternalistic. - Institutions have control responsibility; smaller groups can monitor. - Maintaining language to reflect nuances. | monitoreo de la calidad |
| 26 | desarrollar | develop | **Back translator:** Pretty sure it’s develop, but I wanted to make sure we don’t mean design (diseñar).  **Team:**   - It’s used both ways. In some instances, it is “develop”, other times it is “design”. Do we want to make the instances where it is “design” diseñar instead? - One team member feels like he uses “develop” and “design” interchangeably when talking. We should be fine, but let’s be consistent with distinguishing the two words. - Use “desarrollar” when we mean develop. - Use “diseñar” when we mean design. | desarrollar |
| 18 | necesidades | needs | **Back translator:** I wanted to make sure you didn’t want necessities – it’s fairly interchangeable.  **Team:** Refers to “needs” |  |
| 53 | clínicos | clinicians | **Back translator:** From context, I think all instances referred to clinicians – I wanted to confirm that we did not mean clinics in any instance (centro médico).  **Team:**   - It refers to clinicians in all instances unless it’s just “clinical” as the adjective. - Is there a way to denote individuals in some?– Concerns it might be confused with organization. - Alternative potential suggestion: trabajadores clínicos - Make it consistent so clínicos is used uniformly. | clínicos |
| 35 | impulsar | drive | **Back translator:** Does this mean: Drive? Boost? Push for?  **Team:** Drive is the word in the original. The translation is fine. | impulsar |
| 48 | tiempo protegido | protected time | **Back translator:** Do you mean dedicated time? (tiempo dedicado)  **Team:** Protected time in original | tiempo protegido |
| 52 | tejido de redes sociales | knitting/weaving of social networks | **Back translator:** I can get an idea of what this means, but I can’t think of how to translate it. The most literal is knitting/weaving, but I think the idea is more along the lines of intertwining.  **Team:**   - “Network weaving” is the term used in the original. Team agrees that feels like a literal translation. - Team doesn’t know what this means in English. - If it means ‘connecting of social networks’, alternative potential suggestion is: conectar tejido social - Construir tejidos social?- This feels like this is creating something new, but the original is taking what’s already existing. - Alternative potential suggestion: Promover tejido social - Alternative potential suggestion: Conectar redes sociales | conectar redes sociales |
| 23 | capacitación | training | **Back translator:** Training vs capacitation  **Team:** The word refers to training, such as educating about the innovation. | capacitación |
| 16 | divulgación educativas | dissemination of information | **Back translator:** I don’t know exactly how to translate in this context – I've never heard it like this before.  **Team:**   - “Educational outreach” is the phrase in the original. - Alternative potential suggestion: “de extension.” - The translation feels different than original. - Team suggests use of extension | extensión educativa |
| 64 | personas interesadas | interested parties/persons | **Back translator:** I defaulted to “interested persons/people” for the translation to differentiate between the use of “personas” and “partes.” It’s a more literal translation, but  interested parties can be used interchangeably as that is what’s understood by the statement.  **Team:**   - We went back and forth with concept this in the forward translation. It’s “stakeholders” in the original. - Personas is group of people. - Partes could be things that are not just people. - Using one consistent phrase may make the most sense- just use “partes” to be more comprehensive. - Alternative potential suggestion to use word for “partners?” - Implementation science community is steering away from the use of the word “stakeholder.” - “Partes interesadas” would be more inclusive. | partes interesadas |
| 69 | medios de comunicación en masas | communication mediums/media in mass | **Back translator:** Does this mean mass media? - (Medios de comunicacion masivos)  **Team:**   - “Mass media” is the phrase in the original. - Some of the team prefers back translator’s suggestion to “comunicación masivos.” - The edit may be a dialect thing, but the translations are capturing the same idea. - Use “masivos” to be more generalizable. | medios de comunicación masivos |
| 66 | pagos capitados | capped payments | **Back translator:** I couldn’t think of a way to translate this until I looked at the description – usar pagos fijos? (fixed payments)  **Team:**   - The team doesn’t exactly know what this concept means in English. - It’s “capitated payments” in the original document. - Capitated means something different. - Is this is a concept that doesn’t exist in other contexts? - “Pagos fijos” feels more face valid. - The ancillary materials are helpful for understanding this concept. - Add the ancillary materials to the definition for translation. | pagos fijos |
| 11 | el equipo | equipment | **Back translator:** I understand this as equipment based on context, but this could mean team (Equipamiento?).  **Team:**   - “Equipment” is the word in the original. - The translation is fine. - Context clues help to differentiate words. | el equipo |

**^a^**The phrase may appear in multiple ERIC strategies, so reference is not exhaustive of all occurrences.
